# Four-Week Outcomes of Transforaminal Epidural Injections in Patients With Lumbar Radicular Pain

**DOI:** 10.7759/cureus.75929

**Published:** 2024-12-18

**Authors:** Syed Hassan Raza Bokhari, Muhammad Zarak Awais, Muhammad Tahir Iqbal

**Affiliations:** 1 Orthopedic Surgery, King Edward Medical University, Lahore, PAK

**Keywords:** epidural, lumbar, pain, transforaminal, visual analog scale

## Abstract

Background

Lumbar radicular pain occurs due to irritation or compression of the nerve roots in the lower back. This study aimed to evaluate the efficacy of transforaminal epidural steroid injections (TFESIs) in reducing pain and improving functional outcomes in a diverse patient population with lumbar radicular pain.

Methodology

This quasi-experimental trial was performed at the Department of Orthopedic Surgery and Traumatology, Unit 1, Mayo Hospital, Lahore, from October 2021 to September 2022. The inclusion criteria comprised patients of either gender experiencing lumbar radicular pain. The demographic and clinical profiles of all participants were recorded using a structured questionnaire. Functional status was assessed using the Oswestry Disability Index (ODI). Pain intensity was evaluated using the Visual Analog Scale (VAS). Lumbar TFESI were administered, consisting of 4 mg dexamethasone and 0.33% lidocaine in a 3 ml solution. Patients were monitored for four weeks post-treatment, and outcomes were reported in the form of pain score (VAS) and ODI score from baseline to four weeks. Secondary outcomes included the rate of return to work, patient satisfaction, and the need for additional analgesic use.

Results

Of the total 62 patients, 37 (59.7%) were female. The mean age was 53.34±6.84 years. The mean duration of pain was 2.31±0.95 months. Six patients lost follow-up, so those were excluded from the final analysis. The mean pain score significantly reduced from 7.0±0.85 to 1.90 ± 0.65 after four weeks of treatment (p<0.0001). There was a corresponding improvement in ODI scores from 38.5±8.7 to 20.1±5.3 (p<0.0001). Post-treatment, 38 (61.4%) patients were able to return to work within four weeks. Overall, 49 (87.5%) patients reported satisfaction with the treatment. The requirement for rescue analgesia post-injection was significantly lower, with nine (16.1%) patients needing additional pain relief measures (p<0.0001).

Conclusion

This study demonstrated that lumbar TFESI were effective in significantly reducing pain and improving functional outcomes after a four-week period in patients with lumbar radicular pain.

## Introduction

Back pain is the most common chronic pain disorder in the adult population [1]. In the general population, the burden of low back pain ranges between 4.2-80% [2]. Lumbar radicular pain occurs due to irritation or compression of the nerve roots in the lower back. It affects approximately 13% to 40% of individuals at some point in their lives [3]. The most frequent underlying causes include disc herniation and narrowing of the spinal canal, known as spinal stenosis [4].

Various non-surgical methods can also be exercised that have been used to treat lumbar radicular pain since ancient times [5]. These methods include manipulations, conventional physiotherapy, and other traction methods. However, these traditional methods led to an additional burden on healthcare practitioners and surgeons [6]. Advancements in the field of medical sciences have also led to the development of epidural analgesia that can be used to treat this condition as well. Epidural steroids have been used extensively for prolapsed lumbar disc management [7]. Epidural injections can be administered in one of the three ways including interlaminar, caudal, and transforaminal [8]. Caudal administration also gives effective results in the management of lumbar disc prolapse. Data has revealed that epidural injections often lead to surgical avoidance [9,10]. A transforaminal lumbar epidural injection is given in epidural space by exiting nerve root also known as the foramen. These injections are also known as root blocks or root sleeve blocks [11,12]. One trial showed that the mean pain score at baseline was 8.8±1.4, which was reduced to 1.6±0.8 with transforaminal epidural injection. The mean reduction was 7.2±0.6 after four weeks of treatment, and the difference was statistically significant [13].

Despite the availability of various treatment options, achieving optimal pain relief and functional recovery remains challenging. Lumbar transforaminal epidural steroid injections (TFESIs) are commonly used to manage this condition, but there is variability in patient response and limited data on the effectiveness of TFESIs in different demographic and clinical subgroups. This study aimed to evaluate the efficacy of TFESIs in reducing pain and improving functional outcomes after a four-week period in a diverse patient population with lumbar radicular pain. By analyzing the impact of these injections on pain intensity, functional status, and the need for additional pain relief, this research sought to provide evidence-based guidance on the use of TFESIs in clinical practice.

## Materials and methods

This quasi-experimental study was conducted at the Department of Orthopedic Surgery and Traumatology, Unit 1, Mayo Hospital, Lahore, from October 2021 to September 2022. The inclusion criteria comprised patients of either gender experiencing lumbar radicular pain. Exclusion criteria were patients with a history of spinal fracture or previous spinal surgery, recurrent pain within three months of prior treatment, those on steroid therapy, and individuals with known allergies to the trial medications. The study was registered under trial registration number NCT06656429. Approval from the Institutional Ethical Committee of Kind Edward Medical University/Mayo Hospital, Lahore, Pakistan was acquired (Letter number: KEMU/2022/RC/65). Informed and written consents were obtained. A sample size of 62 was calculated considering the proportion of low back pain in the general population at 4.2% [2], with a 95% confidence level and 5% margin of error.

The demographic and clinical profiles of all participants were recorded using a structured questionnaire, capturing variables such as age, gender, body mass index (BMI), duration and history of pain, comorbidities (e.g., hypertension, diabetes, osteoarthritis), prior use of analgesics and nonsteroidal anti-inflammatory drugs (NSAIDs), and specific vertebral involvement. Neurological examination findings, including motor strength and sensory deficits, were documented. Functional status was assessed using the Oswestry Disability Index (ODI).

Pain intensity was evaluated using the Visual Analog Scale (VAS), where patients rated their pain on a scale from 0 (no pain) to 10 (worst pain). Lumbar TFESIs were administered, consisting of 4 mg dexamethasone and 0.33% lidocaine in a 3 ml solution, under fluoroscopic guidance and a paraspinal approach. The TFESI was aimed toward the proximal and lower edge of the transverse process, checking in with both projections and confirmed with contrast before injecting. All procedures were performed in the operation room. Patients were monitored for four weeks post-treatment, with follow-up visits scheduled at one week and four weeks. Pain scores were reassessed using VAS and functional improvement was measured using the ODI at the end of the follow-up period. Patients lost to follow-up were excluded from the final outcome analysis. The primary outcome measures were the change in VAS pain score and ODI score from baseline to four weeks. Secondary outcomes included the rate of return to work, patient satisfaction, and the need for additional analgesic use.

Data were analyzed using IBM SPSS Statistics, version 26.0 (IBM Corp., Armonk, USA). Categorical data were presented as frequencies and percentages, while continuous data were expressed as mean ± standard deviation (SD). Paired t-tests were performed to compare baseline and post-treatment scores. For categorical data, a chi-square test was applied for the comparison. A p-value of less than 0.05 was considered statistically significant.

## Results

Of the total 62 patients, 37 (59.7%) were female. The mean age was 53.34±6.84 years, with 38 (61.3%) patients aged between 51-80 years. The mean BMI was 29.5±3.21 kg/m². The mean duration of pain was 2.31±0.95 months. Medical history of hypertension, diabetes, and osteoarthritis were present in 18 (29.0%), 12 (19.4%), and six (9.7%) patients, respectively. Previous use of analgesics was noted in 40 (64.52%) patients, while 22 (35.48%) were taking NSAIDs. No patients reported a history of opioid use prior to the study. The mean ODI score was 38.5±8.7, indicating moderate disability. MRI findings revealed that 40 (64.52%) patients had disc herniation, while 22 (35.48%) had lumbar spinal stenosis. The most commonly affected vertebrae were L4-L5 (58.06%) and L5-S1 (32.25%). A neurological examination indicated motor weakness in 18 (29.03%) patients, with sensory deficits present in 24 (38.71%) patients. Table 1 shows the baseline demographic and clinical characteristics of the patients. The mean VAS pain score at baseline was recorded as 7.0±0.85.

**Table 1:** Baseline and demographic characteristics (n=62) ODI: Oswestry Disability Index; NSAIDs: Nonsteroidal anti-inflammatory drugs

Variables		Frequency (%)	Mean±SD
Gender	Male	25 (40.3%)	-
	Female	37 (59.7%)	-
Age (years)	20-50	24 (38.7%)	-
	51-80	38 (61.3%)	-
Age (years)		-	53.34±6.84
Body mass index (kg/m^2^)		-	29.50±3.21
Duration of pain (months)		-	2.31±0.95
Comorbidites	Hypertension	18 (29.0%)	-
	Diabetes	12 (19.4%)	-
	Osteoarthritis	6 (9.7%)	-
Medical history	Prior analgesics use	40 (64.5%)	-
	Prior NSAIDs use	22 (35.5%)	-
Functional assessment (ODI score)		-	38.56±8.73

Six patients lost follow-up, so those were excluded from the final analysis. The mean pain score significantly reduced to 1.90±0.65 after four weeks of treatment (p<0.0001). There was a corresponding improvement in ODI scores from 38.5±8.7 to 20.1±5.3 (p<0.0001). Patients with disc herniation showed a significant reduction in pain scores (6.9±0.7 to 1.5±0.4, p<0.001), and the same was reported in patients with spinal stenosis (7.2±0.9 to 2.3±0.7, p=0.002). Post-treatment, 38 (61.4%) patients were able to return to work within four weeks. Overall, 49 (87.5%) patients reported satisfaction with the treatment. The requirement for rescue analgesia post-injection was significantly lower, with nine (16.1%) patients needing additional pain relief measures (p<0.0001). No serious complications were reported. Minor complications included transient numbness in three (5.4%) and mild headache in two (3.6%) patients. The details about the comparison of pain and functional outcomes at baseline and after four weeks of treatment are shown in Table 2.

**Table 2:** Comparison of pain and functional outcomes VAS: Visual Analog Scale

Outcomes	Baseline (n=62) (Mean±SD)	After four week (n=56) (Mean±SD)	T-statistics/Chi-square value	P-value
Pain score (VAS)	7.0±0.85	1.90±0.65	-36.32	<0.0001
ODI score	38.5±8.7	20.1±5.3	-13.69	<0.0001
Return to work	-	38 (61.4%)	-	-
Patient satisfaction	-	49 (87.5%)	-	-
Analgesic use	40 (64.5%)	9 (16.1%)	28.44	<0.0001

## Discussion

The present study aimed to evaluate the efficacy of lumbar TFESIs in reducing pain and improving functional outcomes in patients presenting with lumbar radicular pain. Our results demonstrated a significant reduction in both pain intensity and disability, as measured by the VAS and the ODI, respectively. The mean VAS pain score decreased from 7.0±0.85 at baseline to 1.90±0.65 after four weeks of treatment (p<0.0001), and the ODI score improved from 38.5±8.7 to 20.1±5.3 (p<0.0001). These findings suggest that TFESI is an effective intervention for managing lumbar radicular pain and restoring functional capacity in this patient population. Hashemi et al. reported significant pain reduction following TFESI in patients with lumbar disc protrusion, with the mean VAS decreasing from 6.91 to 4.67 (p=0.002) [14]. Similarly, the ODI score improved from 47.23 to 37 (p<0.001). While our study showed a greater reduction in pain and disability, this difference may be attributed to variations in patient demographics, duration of symptoms, and differences in the corticosteroid dosages used. Suleiman et al. also demonstrated the efficacy of TFESI in a prospective observational study, where the mean baseline pain and ODI scores decreased significantly at six months post-intervention [15]. However, their study used triamcinolone acetonide combined with bupivacaine, whereas our study utilized dexamethasone and lidocaine. This difference in corticosteroid formulation may have contributed to variations in the observed outcomes as the pharmacodynamics of these agents can differ. The clinical implications of our study are significant, particularly in light of the high prevalence of lumbar radicular pain, which affects up to 40% of individuals during their lifetime. The substantial reduction in pain and improvement in functional outcomes observed in our study suggest that TFESIs can be considered a preferred non-surgical intervention for patients with lumbar radicular pain. Importantly, 61.4% of patients were able to return to work within four weeks of receiving TFESI, which underscores the potential of this treatment to improve not only clinical but also socioeconomic outcomes. Furthermore, patient satisfaction was high, with 87.5% of patients expressing contentment with their treatment results. This level of satisfaction is consistent with findings from Kennedy et al. who reported similar high satisfaction rates following TFESI in a multicenter randomized trial comparing dexamethasone and triamcinolone [16].

Our study also revealed that patients with disc herniation showed a more pronounced reduction in pain scores compared to those with spinal stenosis (6.9±0.7 to 1.5±0.4 vs. 7.2±0.9 to 2.3±0.7, respectively). This is consistent with the literature as TFESI tends to be more effective in patients with disc herniation, likely due to its direct action on the inflamed nerve root [17]. MacVicar et al. in their comprehensive review noted that while TFESI is beneficial in cases of contained disc herniations, its effectiveness diminishes in patients with more extensive pathology such as high-grade stenosis or severe nerve root compression [18]. These findings highlight the importance of patient selection in optimizing treatment outcomes.

The requirement for additional analgesic use decreased significantly following TFESI, from 64.5% at baseline to 16.1% post-treatment (p<0.0001). This reduction in medication reliance not only indicates the effectiveness of TFESIs in managing pain but also reduces the risk of adverse effects associated with long-term analgesic use, such as gastrointestinal complications from NSAIDs or dependence on opioids [19]. Our findings are corroborated by those of Haring et al. who reported a similar decrease in pain medication use post-TFESI in a retrospective cohort study [20]. This has important clinical implications, particularly in the context of the ongoing opioid crisis, as it suggests that effective interventional pain management strategies like TFESIs could potentially mitigate the need for opioid prescriptions in this patient population [21].

In terms of safety, no serious complications were reported in our study. Minor complications included transient numbness (5.4%) and mild headache (3.6%), both of which resolved spontaneously. These findings are consistent with those of Munir et al. who reported no severe adverse events in a large cohort of patients receiving TFESI [22]. The overall safety profile of TFESI makes it an attractive option for patients seeking non-surgical pain relief. However, the risk of complications, though minimal, must be communicated to patients as part of the informed consent process.

Our study had several limitations that must be acknowledged. Firstly, the quasi-experimental design lacks randomization and a control group, which could introduce selection bias and limit the generalizability of our findings. Future studies utilizing a randomized controlled design are needed to confirm our results. Secondly, the follow-up period was limited to four weeks, which may not capture the long-term efficacy and potential recurrence of symptoms. Thirdly, the sample size, though adequate for detecting significant changes in pain and ODI scores, may not be sufficient to identify less common adverse events or differences in response among various subgroups, such as those with different comorbidities or anatomical variations. Future research should explore the comparative effectiveness of different corticosteroid formulations in TFESIs to optimize treatment protocols.

## Conclusions

This study demonstrated that lumbar TFESIs were effective in significantly reducing pain and improving functional outcomes after a period of four weeks in patients with lumbar radicular pain. The treatment was associated with high patient satisfaction and a favorable safety profile. Future studies should focus on optimizing patient selection criteria, exploring the long-term outcomes of TFESIs, and comparing different corticosteroid formulations to further refine this therapeutic approach.
